# Seroepidemiological Investigation and Risk Factors of Schmallenberg Virus Infection in Sheep and Goats in Bangladesh

**DOI:** 10.1155/tbed/5788478

**Published:** 2026-04-30

**Authors:** Ariful Islam, Md Abu Sayeed, Monjurul Islam, Md. Kaisar Rahman, Khondoker Shahriar Islam, Hameem Mollick Meem, Josefina Abedin, Anowar Hossen, Abdul Ahad, Shariful Islam, Jade K. Forwood

**Affiliations:** ^1^ Gulbali Research Institute, Charles Sturt University, Wagga Wagga, 2678, New South Wales, Australia, csu.edu.au; ^2^ National Centre for Epidemiology and Population Health (NCEPH), Australian National University, Canberra, 2601, ACT, Australia, anu.edu.au; ^3^ Zoonotic Disease Research Program, Institute of Epidemiology, Disease Control and Research (IEDCR), Mohakhali, Dhaka, 1212, Bangladesh, iedcr.org; ^4^ Department of Microbiology and Veterinary Public Health, Chattogram Veterinary and Animal Sciences University, Chattogram, 4225, Bangladesh, cvasu.ac.bd; ^5^ Queensland Alliance for One Health Sciences, School of Veterinary Science, The University of Queensland, Brisbane, 4343, Queensland, Australia, uq.edu.au; ^6^ Transboundary Animal Diseases Research Center, Bangladesh Livestock Research Institute, Savar, Dhaka, 1341, Bangladesh, blri.gov.bd

**Keywords:** risk factors, schmallenberg virus, seroprevalence, small ruminants, transboundary infection, vector-borne disease

## Abstract

Schmallenberg virus (SBV) is an emerging vector‐borne pathogen that significantly impacts the health and productivity of both domesticated and wild ruminants, leading to considerable economic losses. Its transmission via arthropod vectors raises concerns about potential geographic expansion, particularly to South Asia, including Bangladesh, where livestock farming plays a vital role in rural livelihoods. Hence, this study aimed to estimate the serological evidence of SBV exposure and identify associated risk factors in small ruminants in Bangladesh from January 2017 to June 2019. Individual animal characteristics were recorded using a structured questionnaire. A total of 517 serum samples were collected from randomly selected goats (*n* = 230) and sheep (*n* = 287) across three districts: Dhaka, Chattogram, and Faridpur, representing both market and household settings. Serum samples were screened using a commercial indirect multi‐species ELISA to detect antibodies against the SBV nucleoprotein. The overall seroprevalence of SBV was 19.5% (101/517, 95% CI: 16.2–23.2), with sheep showing higher seropositivity at 30.0% (86/287, 95% CI: 24.7–35.6) compared to goats at 6.5% (15/230, 95% confidence interval [CI]: 3.7–10.5). In a multivariable logistic regression model, sheep had significantly higher odds of SBV seropositivity than goats (odds ratio [OR]: 6.4; 95% CI: 3.6–12.2; *p*  < 0.01). Animals drinking from pond water sources also had a greater risk than those using supplied water (OR: 2.3; 95% CI: 1.2–4.5; *p* = 0.01). This study provides the first serological evidence of SBV exposure in small ruminants in Bangladesh. The findings underscore the need for targeted surveillance and improved biosecurity and management practices to reduce the risk of SBV transmission in the region.

## 1. Introduction

Schmallenberg virus (SBV), a vector‐borne pathogen, has become a major threat to the health and productivity of both wild and domestic ruminants. SBV is an *Orthobunyavirus* belonging to the family *Peribunyaviridae* and was first identified in Schmallenberg, Germany, in 2011 following reports of acute clinical disease characterized primarily by fever, reduced milk yield, and diarrhea in adult cattle, rather than congenital malformations [1–3]. Subsequent investigations linked SBV infection during pregnancy to congenital malformations in ruminant offspring, which substantially increased its veterinary and economic significance. SBV has demonstrated its ability to spread rapidly across Europe, indicating its significant potential for dissemination and posing serious threats to livestock health [3]. These characteristics underscore the importance of a thorough understanding of SBV epidemiology and transmission dynamics to inform surveillance strategies and mitigate future outbreaks.

SBV has a tripartite, negative‐sense genomic RNA and is primarily transmitted by biting midges (*Culicoides* spp.), which serve as the main vectors for its propagation [2, 3]. The virus can infect a wide range of ruminants, including cattle, sheep, and goats. While adult animals often experience subclinical or mild symptoms such as fever, reduced milk yield, and diarrhea [2, 4], pregnant animals are particularly vulnerable due to the risk of transplacental transmission. Although direct evidence of identifying SBV‐competent Culicoides species in South Asia is currently limited, the region’s favorable geo‐climatic conditions and widespread distribution of Culicoides vectors suggest a potential risk for virus establishment and transmission if SBV were introduced [5]. Beyond ruminants, SBV exposure has been documented through seroconversion in pigs, wild boar, and carnivores, indicating a broader host range than initially recognized [6–8]. Infection during critical stages of pregnancy can lead to severe congenital malformations, including arthrogryposis and hydranencephaly, with the highest vulnerability occurring between days 28 and 56 of gestation in sheep and days 80 and 150 in cattle [2, 9].

Evidence of SBV circulation has recently emerged outside of Europe, including serological evidence of SBV in small ruminants in Malaysia, where infection rates up to 27.8% were reported across multiple Peninsular states, indicating active virus transmission in local small ruminants [10]. The consequences of SBV are multifaceted, extending beyond animal health to threaten rural livelihoods and the sustainability of livestock systems. In addition, SBV or SBV‐like viruses have been reported in livestock in China [11], underscoring the expanding geographic footprint of the virus. Collectively, these findings highlight that SBV poses a multifaceted threat extending beyond animal health to rural livelihoods and the sustainability of livestock production systems, particularly in regions with limited surveillance.

The outbreak of SBV poses considerable economic and production impacts, primarily through treatment costs, production losses, and reduced reproductive performance in commercial flocks. It may also lead to indirect losses due to trade restrictions [12, 13]. The pathogenesis of SBV results in neonatal loss due to abortion and stillbirth, while reduced milk production further undermines herd productivity and flock profitability [4]. Although direct estimates from Bangladesh are lacking, evidence from European settings demonstrates substantial economic burdens associated with SBV, which provides useful context for understanding its potential impact in this country, where livestock farming is largely entwined with the rural economy. Smallholder farmers, who depend on livestock for income, food security, and as a coping strategy during crises, may be particularly vulnerable to emerging infections. The possible introduction of SBV into Bangladesh could further impact the existing livestock production systems, particularly among smallholder farmers.

The emergence and re‐emergence of vector‐borne diseases from endemic regions into novel geographic areas have become a major threat to livestock health, production, and rural livelihoods. Such dynamics also increase the potential for the spread of SBV into previously unaffected regions. In Bangladesh, the existence of other vector‐borne livestock diseases, such as bluetongue virus and lumpy skin disease [14, 15], highlights the country’s vulnerability to similar infections. However, information on the occurrence or circulation of SBV in this country is currently lacking. Although this study did not directly collect environmental or entomological data, it contributes to the ecological understanding of SBV transmission by linking animal‐level seropositivity with host and management characteristics within livestock systems operating in defined agro‐ecological contexts. Therefore, this study was undertaken to quantify the seroprevalence of SBV in small ruminants in Bangladesh and to provide baseline evidence on SBV circulation. This is essential to assess the potential risk of outbreaks and to guide future disease control measures. Furthermore, we also identified key epidemiological risk factors related to SBV seropositivity, which can support the development of country‐specific intervention strategies, including integrated vector management and targeted risk communication strategies in Bangladesh.

## 2. Materials and Methods

### 2.1. Ethical Considerations

All procedures involving animals were conducted by trained veterinarians under proper physical restraint to ensure animal safety and minimize distress. Verbal explanations about the study procedures were provided to animal owners, and written informed consent was obtained prior to sample collection. Ethical clearance was obtained from the Institutional Animal Ethics Committee of CVASU under the Protocol Number CVASU/Dir/(R&E) EC/2015/927.

### 2.2. Study Location, Duration, and Sampled Population

**Figure 1:**
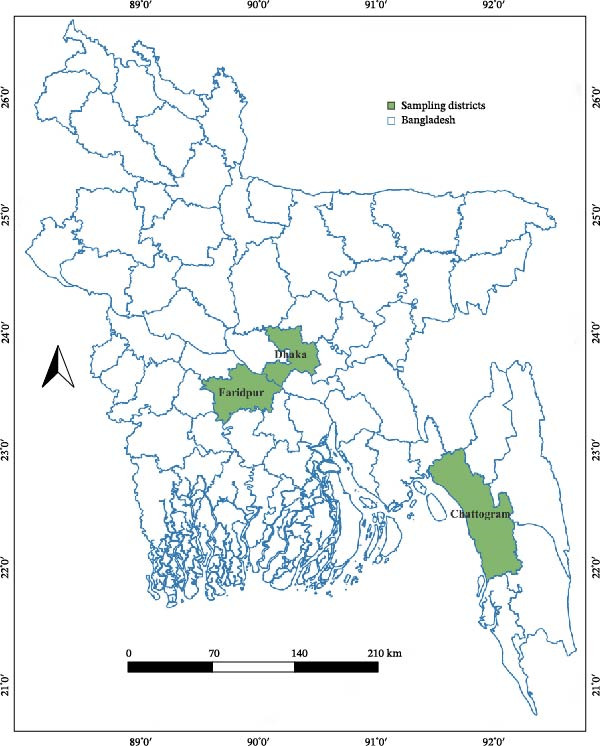
Spatial location of sampling districts in Bangladesh, with district boundaries and sampling sites indicated.

We conducted a cross‐sectional study from January 2017 to June 2019 in three different districts of Bangladesh: Dhaka, Chattogram, and Faridpur. The goat and sheep populations in these districts were collected from the statistics of the agriculture census of the Bangladesh Bureau as follows: Dhaka 217,369 goats and 21,445 sheep; Faridpur 340,499 goats and 4376 sheep; and Chattogram 240,188 goats and 13,713 sheep, respectively [16]. Animals were reared under traditional backyard or smallholder farming systems, where farmers typically keep a few animals for household income and subsistence [16]. The selected districts represent different agro‐ecological zones of Bangladesh. Dhaka and Faridpur are located in the central region, while Chattogram is situated in the southeastern coastal area. Faridpur, Dhaka, and Chattogram experience a tropical monsoon climate with hot and humid summers, where average daytime temperatures range between 32 and 35°C. Winters are mild and relatively dry, with average lows between 13 and 18°C. Most rainfall occurs from June to September during the monsoon season, while the rest of the year remains warm and comparatively dry with average temperatures around 25–32°C [17]. We selected these districts considering livestock density, ecological diversity, and easy accessibility. Samples were collected from goats and sheep across multiple sub‐locations within these study districts. The GPS coordinates (latitude and longitude) of each sampling point were recorded and later visualized using spatial maps in QGIS (Figure 1).

### 2.3. Sample Size Calculation and Sampling Strategy

We calculated the sample size for the seroprevalence of SBV among small ruminants using the standard formula for prevalence studies using OpenEpi (https://www.openepi.com/Menu/OE_Menu.htm): Sample size, *n* = DEFF × [*Z* ^2^ × *P*(1 − *P*)]/*d* ^2^.

Here, *n* is the required sample size, DEFF = 1, *Z* = 1.96, *P* = expected prevalence, and *d* = desired precision. Due to the absence of prior SBV seroprevalence data, an expected prevalence of 50% was considered with 5% precision, resulting in 384 animals. Considering a 25% nonresponse rate, the expected sample size was increased to 517 animals.

We used a simple random sampling approach across three districts. First, we developed a sampling frame by compiling a list of households and commercial farms rearing sheep and/or goats in consultation with the district livestock offices. Then we randomly selected each farm, but if any farm owner declined to participate, the next farm from the list was approached. Within each participating farm, one animal was randomly selected if the herd size was fewer than 10 animals, and two animals were randomly selected if the herd size was 10 or more. To enhance representativeness and achieve the target sample size, additional animals were also randomly selected from local livestock markets, ensuring adequate geographic coverage and minimizing selection bias.

### 2.4. Sample Collection, Preservation, and Transportation

We collected a total of 517 blood samples from individual goats and sheep in the sampled district. To ensure representation, we randomly selected an individual sheep and goat from backyard farming and the local market in each studied district. Approximately 4–5 mL of blood was aseptically collected from the jugular vein of each animal into sterile vacutainers using disposable syringes by a registered veterinarian. During sampling, animals were observed for general clinical signs such as fever, nasal discharge, oral lesions, lameness, or abortion history as reported by owners; however, all sampled animals appeared clinically healthy at the time of collection. Immediately after collection, all samples were placed in an insulated cold box to maintain the cold chain during the transfer to the field laboratory where the blood samples remained for 1–2 h at room temperature to allow clotting and then centrifuged at 3,000 rpm for 10 min to obtain the serum. After that, the serum was aliquoted with appropriate labeling into two cryovials of 1.5 mL each. In the laboratory the samples were frozen at ‐80°C until further analysis.

### 2.5. Laboratory Analysis

We screened individual serum samples using the commercial ID Screen SBV Indirect Multi‐species ELISA kit (IDvet, Montpellier, France) to detect antibodies against the SBV nucleoprotein according to the manufacturer’s instructions. The optical density (OD) of each well was measured at 450 nm using an ELISA reader (Mindray MR‐96 A). The net OD for each sample was calculated by subtracting the OD of the odd‐numbered well from that of the even‐numbered well. To validate the test, the mean net OD of the negative control (NC) had to be less than 0.350, and the ratio of the net OD of the positive control (PC) to the absolute net OD of the NC had to exceed 3. The percentage of sample‐to‐positive control (SP%) was calculated using the following formula:

SP%=Net OD of SampleNet OD of Positive Control×100

where SP% ≤ 50 was considered negative, 50 < SP% ≤ 60 indicating doubtful, and SP% > 60 was considered positive results for SBV antibodies.

At the recommended cutoff (S/P = 60%), this assay has a reported sensitivity of 97.2% and specificity of 99.8%, with 98.9% concordance compared to the virus neutralization test (VNT).

### 2.6. Questionnaire Design and Data Collection

A pretested structured questionnaire was used to collect detailed animal‐level data. The questionnaire was developed based on an intensive literature review and was administered through face‐to‐face interviews with the farm owners or workers. Detailed data was recorded under both animal and environmental‐level variables. Animal‐level predictors included source of animal, species, breed, age (in months), sex, body condition score (BCS), pregnancy status, insemination history, lactation status, history of reproductive disorders, vaccination status (SBV, PPR, Anthrax, Rabies, and goat pox), and presence or absence of clinical signs.

Environmental‐level predictors were defined as variables describing the geographic setting, land use, and management practices that may influence animals’ exposure to vectors. These included district, landscape, rearing system, grazing system, water source, and vector control practices. We categorized sheep and goats into two groups for sample collection: adults (>6 months of age) and juveniles (<6 months of age) [18]. All data were recorded electronically in a Microsoft Excel spreadsheet.

### 2.7. Statistical Analysis

All field and laboratory data were cleaned using Microsoft Excel 2019. We performed the data analysis in R (version 4.2.0). After importing and cleaning the dataset, relevant variables were selected and forwarded for further analysis. We estimated the seroprevalence with a 95% confidence interval (CI) for each predictor level using the appropriate denominator, regardless of subgroup size. We assigned flock‐ and environment‐level variables to individual animals within each flock and analyzed at the animal level to examine their association with SBV seropositivity. We performed univariable analyses using chi‐square or Fisher’s exact tests, as appropriate, to explore associations between SBV seropositivity and individual explanatory variables. Variables were selected for multivariable analysis based on univariable screening (*p* < 0.30) and a priori biological relevance. A total of 12 variables (seven individual and five environmental) were included in the multivariable logistic regression model, except those applicable only to females (pregnancy, insemination, lactation, and reproductive disorder). The analysis was performed using a backward elimination approach. Potential confounding was assessed by examining changes in the estimated coefficients of the main independent variables when adding potential confounders; variables that changed the coefficient substantially (≥10%) were considered confounders and retained in the multivariable model [19]. To check for multicollinearity, we calculated Cramér’s V between variable pairs (Figure S1). Variables showing high collinearity were excluded from the multivariable model. Model fit was assessed using the Akaike information criterion (AIC), with lower AIC values indicating improved relative model fit among competing model specifications. Overall model adequacy was further evaluated using standard goodness‐of‐fit diagnostics. Internal validity was assessed through examination of model diagnostics, coefficient stability during model reduction, and consistency of effect estimates across model specifications. Finally, we performed multivariable logistic regression to identify independent predictors of SBV seropositivity. The results were expressed using odds ratios (ORs) and 95% CI, and statistical significance was considered at *p*  < 0.05.

## 3. Results

### 3.1. Seroprevalence of SBV by Host Factors (Species, Breed, Age, and Sex)

**Figure 2:**
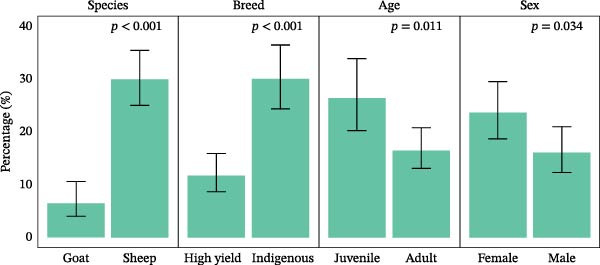
Seroprevalence of SBV by demographic factors within animal categories.

The overall seroprevalence of SBV was 19.5% (101/517, 95% CI: 16.2–23.2). In case of univariable analysis, within the species, sheep showed significantly higher seropositivity at 30.0% (86/287, 95% CI: 24.7–35.6), compared with goats at 6.5% (15/230, 95% CI: 3.7–10.5). Breed was also significantly associated with seroprevalence (*p*  < 0.001), with indigenous animals showed higher antibody prevalence at 30.1% (66/219, 95% CI: 24.1–36.7), than the high‐yielding breed at 11.7% (35/298, 95% CI: 8.3–16.0). Age was another significant determinant (*p* = 0.011), with juvenile animals showing higher seropositivity at 26.4% (42/159, 95% CI: 19.7–34.0), compared to adults at 16.5% (59/358; 95% CI: 12.8–20.7) (Figure 2 and Table 1).

**Table 1:** Univariable logistic regression between different predictors and SBV seropositivity in sheep and goats.

Predictors	Category	Negative (%)	Positive (%)	Positive (95% CI)	*p*‐Value
District	Chittagong	239 (84.2%)	45 (15.9%)	11.8–20.6	0.058
	Dhaka	144 (75.4%)	47 (24.6%)	18.7–31.3	
	Faridpur	33 (78.6%)	9 (21.4%)	10.3–36.8	
Landscape	Peri‐urban	23 (53.5%)	20 (46.5%)	31.2–62.3	<0.01
	Rural	33 (78.6%)	9 (21.4%)	10.3–36.8	
	Urban	360 (83.3%)	72 (16.7%)	13.3–20.5	
Age	Adult	299 (83.5%)	59 (16.5%)	12.8–20.7	0.009
	Juvenile	117 (73.6%)	42 (26.4%)	19.7–34	
Sex	Female	177 (76.3%)	55 (23.7%)	18.4–29.7	0.031
	Male	239 (83.9%)	46 (16.1%)	12.1–20.9	
Source	Commercial farm	110 (82.7%)	23 (17.3%)	11.3–24.8	0.596
	Household farm	231 (80.5%)	56 (19.5%)	15.1–24.6	
	Market	75 (77.3%)	22 (22.7%)	14.8–32.3	
Species	Goat	215 (93.5%)	15 (6.5%)	3.7–10.5	<0.01
	Sheep	201 (70.0%)	86 (30.0%)	24.7–35.6	
Breed	High yield	263 (88.3%)	35 (11.7%)	8.3–16	<0.01
	Indigenous	153 (69.9%)	66 (30.1%)	24.1–36.7	
Pregnancy	No	135 (78.5%)	37 (21.5%)	15.6–28.4	0.183
	Yes	42 (70.0%)	18 (30.0%)	18.8–43.2	
Insemination	Natural	172 (75.8%)	55 (24.2%)	18.8–30.3	0.595
	No	5 (100.0%)	0 (0%)	0–52.2	
Lactating	No	161 (77.8%)	46 (22.2%)	16.8–28.5	0.126
	Yes	16 (64.0%)	9 (36.0%)	18–57.5	
Reproductive disorder	No	325 (80.5%)	79 (19.6%)	15.8–23.8	0.984
	Yes	91 (80.5%)	22 (19.5%)	12.6–28	
Clinical sign	No	289 (81.4%)	66 (18.6%)	14.7–23	0.423
	Yes	127 (78.4%)	35 (21.6%)	15.5–28.7	
BCS	Moderate	381 (80.0%)	95 (20.0%)	16.5–23.8	0.409
	Poor	35 (85.4%)	6 (14.6%)	5.6–29.2	
Rearing system	Intensive	234 (83.0%)	48 (17.0%)	12.8–21.9	0.114
	Semi‐extensive	182 (77.5%)	53 (22.6%)	17.4–28.4	
Grazing system	Continuous grazing	177 (78.0%)	50 (22.0%)	16.8–28	0.206
	Zero grazing	239 (82.4%)	51 (17.6%)	13.4–22.5	
Water source	Deep tubewell	326 (82.5%)	69 (17.5%)	13.9–21.6	<0.01
	Pond	56 (65.9%)	29 (34.1%)	24.2–45.2	
	Supplied	34 (91.9%)	3 (8.1%)	1.7–21.9	
Vaccination	No	227 (77.2%)	67 (22.8%)	18.1–28	0.032
	Yes	189 (84.8%)	34 (15.3%)	10.8–20.6	
Vector control	No	294 (79.9%)	74 (20.1%)	16.1–24.6	0.606
	Yes	122 (81.9%)	27 (18.1%)	12.3–25.3	

### 3.2. Seroprevalence of SBV by Reproductive Factors

**Figure 3:**
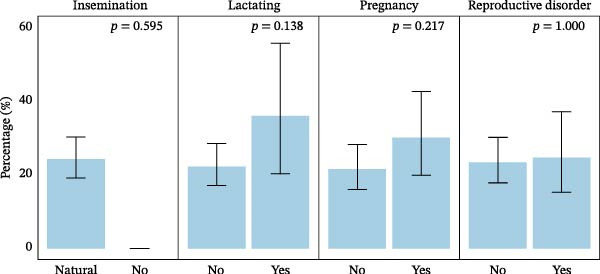
Seroprevalence of SBV by reproductive status within animal categories.

The seroprevalence of SBV did not differ significantly among animals with different reproductive statuses (Figure 3). Seropositivity was slightly higher in animals conceived through natural insemination compared with those with no recorded insemination, though the difference was not significant (*p* = 0.595). Similarly, pregnant animals had marginally greater antibody prevalence (30.0%; 95% CI: 18.8%–43.2%) compared to nonpregnant ones (21.5%; 95% CI: 15.6%–28.4%), while lactating animals also showed a higher but nonsignificant seropositivity (36.0%; 95% CI: 18.0%–57.5%) than nonlactating animals (22.2%; 95% CI: 16.8%–28.5%) (Figure 3 and Table 1).

### 3.3. Univariable Associations Between Animal and Environment Level Predictors and SBV Seropositivity

There were strong correlations between SBV seropositivity and some of the predictors. Seropositivity varied by landscape (*p*  < 0.01). Peri‐urban areas showed the highest seropositivity (46.5%; 95% CI: 31.2%–62.3%), followed by those from rural (21.4%; 95% CI: 10.3%–36.8%) and urban areas (16.7%; 95% CI: 13.3%–20.5%). Ponds showed a significantly higher seroprevalence (34.1%; 95% CI: 24.2%–45.2%) compared to those using deep tube wells (17.5%; 95% CI: 13.9%–21.6%) or supplied water (8.1%; 95% CI: 1.7%–21.9%) (*p*  < 0.01). Similarly, animals without a vaccination history had a higher seroprevalence (22.8%; 95% CI: 18.1%–28.0%) than vaccinated animals (15.3%; 95% CI: 10.8%–20.6%) (*p* = 0.032) (Table 1).

### 3.4. Risk Factors Associated With the SBV Seropositivity Using Regression Model

In the multivariable logistic regression model, species and water source were significantly associated with SBV seropositivity. The odds of sheep being SBV seropositive were 6.4 times higher than the odds for goats (*p*  < 0.01). Animals whose primary water source was pond water had significantly higher odds of SBV seropositivity compared to animals with supplied water (OR: 2.3; 95% CI: 1.2–4.5; *p* = 0.01). Other factors, including sex, age, body condition score, and vaccination status, were not significantly associated with SBV seroprevalence (Table 2).

**Table 2:** Multivariable logistic regression analysis showing the association between selected predictors and SBV seropositivity.

Predictors	Category	Odds ratios (OR)	95% CI	*p*‐Value
Species	Goat	Ref	—	—
	Sheep	6.4	3.6–12.2	<0.01
Sex	Male	Ref	—	—
	Female	1.2	0.8–2.0	0.33
Age	Adult	Ref	—	—
	Young and suckler	1.3	0.8–2.2	0.34
BCS	Poor	Ref	—	—
	Moderate	1.8	0.7–5.1	0.23
	Good	2.2	0.8–6.5	0.14
Vaccination	No	Ref	—	—
	Yes	1.1	0.6–1.9	0.72
Water source	Deep tube well and supply	Ref	—	—
	Pond	2.3	1.2–4.5	0.01

## 4. Discussion

This study demonstrates the presence of SBV‐specific antibodies among small ruminants in Bangladesh and provides new insights into potential epidemiological risk factors associated with seropositivity. This finding broadens the known geographic distribution of SBV beyond Europe and the Middle East, suggesting that tropical and subtropical regions of South Asia may also support the virus’ transmission cycle. This study indicates that SBV is likely circulating among goat and sheep populations of this country and underscores the necessity for increased awareness and further surveillance. The existence of competent vector species (*Culicoides* spp.), coupled with favorable environmental conditions, and close interactions between livestock species may facilitate local SBV transmission [3, 20, 21]. The transmission of SBV is highly sensitive to climatic suitability for the survival and proliferation of Culicoides, which has been documented in Europe [22], Iran [21], Türkiye [23], and sub‐Saharan Africa [24].

The seroprevalence of SBV was significantly associated with species and water source variables. Notably, seroprevalence is higher in sheep compared to goats, a finding consistent with prior studies that suggest sheep are more clinically prone to SBV infection [25, 26]. This also aligns with several studies conducted in Germany, the Netherlands, and France reporting that sheep experienced higher morbidity and mortality than goats during several outbreaks between 2011 and 2013 [22, 27, 28]. Although age was not a significant risk factor, juvenile animals also had a higher prevalence than adult animals. This pattern differs from reports from Iran and Türkiye, where adult animals were more frequently infected [21, 23]. A similar age‐related trend suggesting that adults accumulate antibodies through repeated annual exposure to infected midges was observed in Poland, Germany, and the United Kingdom [20, 29–31].

Maternal immunity may partially explain why younger animals in this study showed lower seropositivity, despite ongoing SBV circulation. Passive transfer of antibodies from naturally infected dams to offspring has been documented in lambs, and studies suggest that maternally derived antibodies can persist for several months, potentially delaying seroconversion following viral exposure [32, 33]. As our study did not assess maternal antibody levels, seroprevalence among young animals may have been underestimated, leading to a misinterpretation of age‐related infection patterns. This highlights the importance of incorporating maternal immunity assessment into future SBV research, particularly when interpreting serological findings in youngstock populations.

In Bangladesh, a semi‐extensive sheep production system may increase the SBV exposure risk due to greater outdoor vector contact, as supported by another study conducted in Spain [34]. However, the overall seroprevalence (19.5%) in the current study is lower than those observed in Spain (29.8%) and Poland (37.5%) [30, 35], but higher than in Türkiye [23]. These variations may be due to differences in climatic conditions, vector seasonality, animal density, and diagnostic approaches used across studies. In Europe, seasonal peaks of Culicoides activity and high livestock movement during summer facilitated widespread outbreaks, whereas Bangladesh’s tropical climate may maintain low‐level year‐round transmission without explosive epidemics. Additionally, regional livestock trade between Bangladesh, India, and Myanmar could serve as a plausible pathway for viral introduction as reported for other vector‐borne pathogens like bluetongue and PPR.

In contrast to the temperate climate of Europe where SBV was first detected, the tropical climate of Bangladesh may allow vectors to remain active throughout the year, thereby extending the potential transmission period. SBV is primarily transmitted by Culicoides biting midges, whose ecology is closely linked to climatic factors such as temperature, rainfall, and humidity. While the specific Culicoides species responsible for SBV transmission in Bangladesh remains unknown, these midges typically breed in moist, organic environments, including muddy soils, livestock manure, irrigated agricultural land, and areas with standing water. In Bangladesh, the monsoon season, high ambient humidity, and widespread livestock–wetland interfaces create ideal conditions for vector breeding and survival [36]. These environmental characteristics likely contribute to regional differences in SBV epidemiology compared to temperate settings. The reporting of year‐round transmission potential of Culicoides‐borne viruses such as bluetongue and Akabane in Southeast Asia further supports the likelihood of persistent SBV exposure in tropical regions [37]. Additionally, SBV circulation has been documented in countries within the broader region, with confirmed detection reported from parts of China, including Guangdong Province [11]. More recently, widespread serological evidence of SBV infection has been identified in sheep and goat populations across multiple states in Peninsular Malaysia, with seroprevalence reaching nearly 28%, indicating ongoing and active transmission among small ruminants [10].

Host‐related factors such as breed susceptibility and immune response may also explain the observed variations in SBV seroprevalence. In Bangladesh, although goats are mostly reared under semi‐intensive systems, indigenous sheep are typically raised under open grazing conditions, increasing their exposure to vector habitats [38]. This observation aligns with previous studies reporting higher SBV seroprevalence among extensively grazed ruminants [35, 39]. Moreover, Culicoides species are associated with intensive farming environments; vector exposure is also shaped by host availability, animal density, and vector host preference rather than landscape type alone. On the other hand, genetic differences and immune competence among breeds may also influence SBV seroprevalence; however, this pattern was not observed in our study. Experimental infection studies have shown that European sheep breeds showed stronger antibody responses than local breeds [40].

Additionally, the 6‐year serosurvey from Poland showed that SBV seroprevalence in ruminants fluctuated across years, with sentinel youngstock testing positive annually, indicating sustained low‐level transmission throughout the year [30]. This pattern may mirror the situation in Bangladesh, where the absence of clinical cases could mask persistent subclinical exposure among ruminants. Such low but steady seropositivity levels are typical of endemic vector‐borne infections in tropical climates, where transmission intensity is balanced by herd immunity and climatic consistency [22].

The interpretation of serological data must consider potential diagnostic limitations. Cross‐reactivity with other orthobunyaviruses, such as Akabane and Aino viruses, which are endemic in parts of Asia, could lead to false positives [41]. Although the ELISA used in this study targets the recombinant nucleoprotein (N protein) and has high specificity, confirmatory molecular techniques such as RT‐PCR and virus isolation remain necessary to validate true infection status [42]. Therefore, integrating serological and molecular methods in future studies would provide a more accurate assessment of SBV circulation in Bangladesh. The findings from this study provide a foundation for designing locally adapted disease control strategies, including vector management, changes in grazing practices, and the potential evaluation of vaccination options should a safe and effective vaccine become available. In parallel, awareness‐raising campaigns and training for farmers, veterinarians, and animal health workers will be essential for early recognition and response.

Importantly, this research aligns with national livestock development priorities in Bangladesh, particularly those aimed at reducing reproductive losses in small ruminants and safeguarding rural livelihoods. Incorporating SBV surveillance into existing systems for monitoring transboundary and vector‐borne diseases could offer a cost‐effective and sustainable strategy, contributing to long‐term resilience in the livestock sector and supporting broader livestock productivity.

This study has several limitations that should be acknowledged. The sampling was conducted at the individual animal level rather than the flock level, as most farmers in the study area practiced small‐scale backyard livestock rearing, and samples were collected from local markets where animals from multiple flocks were aggregated. Therefore, clustering and sampling weights were not accounted for. Additionally, the assumed 50% expected prevalence for sample size estimation may not fully reflect the true population variability. Maternal antibodies were not measured in lambs and kids. Evidence suggests that passively acquired antibodies can persist for several months in young animals, which may lead to underestimation of seropositivity or misinterpretation of infection status in younger age groups. Again, flock‐ and environment‐level predictors were assigned to individual animals for analysis because only one or two animals were sampled per flock, within‐flock clustering could not be reliably estimated, and multilevel modeling was therefore not feasible. Despite these limitations, the study provides essential baseline data on SBV seroprevalence in the region.

## 5. Conclusions

This study provides the first serological evidence of SBV circulation in small ruminants in Bangladesh, highlighting a notable seroprevalence and identifying key epidemiological risk factors. The findings suggest the silent, likely subclinical spread of SBV in the absence of reported clinical disease and highlight species‐specific susceptibility, particularly in sheep raised under extensive systems. To mitigate this emerging threat, a surveillance strategy encompassing nationwide seromonitoring, vector ecology studies, and molecular diagnostics is warranted. In addition, future research should broaden serological surveys across regions and seasons to capture temporal trends, while parallel entomological investigations are needed to map *Culicoides* spp. species composition, abundance, and vectorial capacity. Integrating such entomological mapping with climate and land‐use data will improve risk prediction and guide targeted vector control. These measures will support early detection, advance understanding of SBV epidemiology in South Asia and inform evidence‐based targeted control strategies, ultimately safeguarding livestock productivity and smallholder livelihoods.

## Author Contributions

**Ariful Islam:** conceptualization, formal analysis, funding acquisition, investigation, methodology, project administration, resources, software, supervision, writing – original draft preparation, writing – review and editing preparation; **Md Abu Sayeed:** data curation, formal analysis, methodology, software, validation, visualization, writing – original draft preparation, writing – review and editing. **Monjurul Islam:** data curation, formal analysis, software, visualization, writing – review and editing preparation; **Md. Kaisar Rahman:** investigation, methodology, validation, writing – review and editing preparation; **Khondoker Shahriar Islam:** investigation, methodology, validation, writing – review and editing preparation; **Hameem Mollick Meem:** investigation, methodology, validation, writing – review and editing preparation; **Josefina Abedin:** investigation, methodology, validation, writing – review and editing preparation; **Abdul Ahad:** methodology, project administration, validation, writing – review and editing preparation; **Shariful Islam:** investigation, methodology, validation, writing – review and editing preparation; **Jade K. Forwood:** project administration, funding acquisition, supervision, writing – review and editing preparation.

## Funding

This study was partially supported by the United States Agency for International Development (USAID) through the Emerging Pandemic Threats PREDICT program (Grant AID‐OAA‐A‐14‐00102). Additional support was provided by the Biosecurity Research Program at the Gulbali Institute and the Training Hub for Regional Industry and Innovation in Virology and Epidemiology (THRIIVE) for A. Islam and J. K. Forwood. Open access publishing facilitated by Charles Sturt University, as part of the Wiley ‐ Charles Sturt University agreement via the Council of Australasian University Librarians.

## Disclosure

The funder had no role in the study design, data collection, analysis, interpretation, or the decision to submit the manuscript for publication.

## Conflicts of Interest

The authors declare no conflicts of interest.

## Supporting Information

Additional supporting information can be found online in the Supporting Information section.

## Supporting information


**Supporting Information** Figure S1: A heatmap of Cramer’s V was used to visualize collinearity among variables. Pairwise Cramer’s V statistics were calculated to assess multicollinearity, and variables demonstrating high collinearity were excluded from the multivariable model.

## Data Availability

The data generated and analyzed during this study are included in the article. Additional raw datasets are available from the corresponding author upon reasonable request.
