# Supplementary figures and images for: Seroepidemiological Investigation and Risk Factors of Schmallenberg Virus Infection in Sheep and Goats in Bangladesh
Source: Transbound Emerg Dis. 2026 Apr 30;2026:5788478. doi: 10.1155/tbed/5788478 (PMC13130852; doi:10.1155/tbed/5788478)

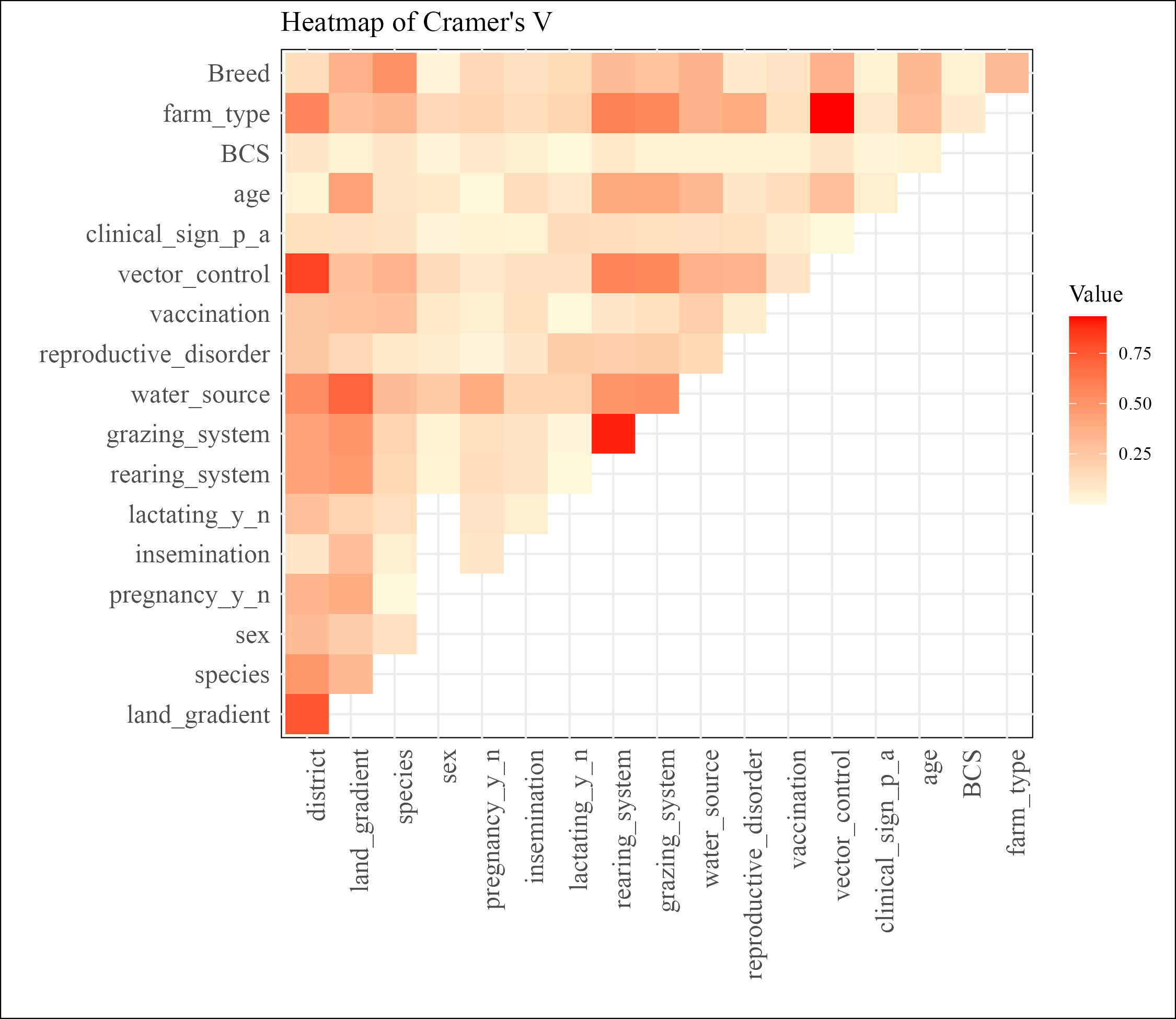


Supplementary Figure 1: Heatmap of Cramer’s V showed the collinearity among variables

Supplement: Supplementary file 1 — Supporting Information Figure S1: A heatmap of Cramer’s V was used to visualize collinearity among variables. Pairwise Cramer’s V statistics were calculated to assess multicollinearity, and variables demonstrating high collinearity were excluded from the multivariable model. [file TBED-2026-5788478-s001.docx]
